# Fatal neural angiostrongyliasis in the Bolivian squirrel monkey (*Saimiri boliviensis boliviensis*) leading to defining *Angiostrongylus cantonensis* risk map at a zoo in Australia

**DOI:** 10.1016/j.onehlt.2023.100628

**Published:** 2023-09-15

**Authors:** Phoebe Rivory, Kresen Pillay, Rogan Lee, David Taylor, Michael P. Ward, Jan Šlapeta

**Affiliations:** a Sydney School of Veterinary Science, Faculty of Science, The University of Sydney, New South Wales 2006, Australia; b Sydney Zoo, Bungarribee, New South Wales 2767, Australia; c NSW Health Pathology, Centre for Infectious Diseases and Microbiology Lab Services, Level 3 ICPMR, Westmead Hospital, Westmead, New South Wales 2145, Australia; d The University of Sydney Institute for Infectious Diseases, New South Wales 2006, Australia; e Vetnostics, North Ryde, New South Wales 2113, Australia

**Keywords:** *Angiostrongylus cantonensis*, Rat lungworm, Neural angiostrongyliasis, Captive primates, *cox*1, mtDNA

## Abstract

Neural angiostrongyliasis (NA) is a parasitic disease caused by *Angiostrongylus cantonensis* (rat lungworm). This study presents a case of NA in a captive Bolivian squirrel monkey from a zoo in western Sydney, Australia. The objective was to identify the *A. cantonensis cox*1 haplotype responsible for the infection and compare its mitochondrial DNA (mtDNA) to known Australian mtDNA. An epidemiological investigation was conducted to assess the risk of infection, focusing on the resident rat population in the zoo. Methods involved trapping rats and collecting rat faeces for *Angiostrongylus* detection, speciation, and *cox*1 haplotype confirmation. Various techniques were employed, including necropsy, morphological examination, and molecular methods such as ITS-2 qPCR, *cox*1 sequencing, and ITS-2 metabarcoding. Cluster analysis of rat faeces distribution and *Angiostrongylus* detection utilised an equal sampling effort (ESE) approach. Gastropods were collected throughout the study for *Angiostrongylus* surveillance using a hypersensitive qPCR assay. Results revealed significant clustering of rat faeces near exhibits with fresh food provision and absence of predators. *Angiostrongylus*-positive faeces were uniformly distributed across the zoo property. Mitochondrial DNA analysis confirmed the presence of the Ac13 haplotype of *A. cantonensis* in the monkey. Morphology, ITS-2 metabarcoding and partial *cox*1 sequencing detected only *A. cantonensis*, with the Ac13 *cox*1 haplotype predominating. A high prevalence of infection (64%, 9/14) was found in brown rats, with quantification of larvae indicating high shedding rates. Co-infections with both Ac13 and local SYD.1 *A. cantonensis cox*1 haplotypes were observed. Only three gastropods (all of which were *Angiostrongylus*-negative) were found in the survey. To minimise the risk of exposure for susceptible species, targeted rodent control was implemented in areas with higher exposure risk. A potential strategy (which requires further exploration) to consider for future zoo design was suggested. This study provides insights into the epidemiology and genetic diversity of *A. cantonensis* in Australia, emphasising the importance of control measures to prevent future outbreaks.

## 1 Introduction

*Angiostrongylus cantonensis,* the rat lungworm, is a metastrongyloid parasite endemic to Southeast Asia and the Pacific Basin, with recent invasions into the US and Europe [[1], [2], [3], [4], [5]]. This nematode can cause potentially fatal neural angiostrongyliasis (NA) in non-permissive “accidental” hosts, including humans [1,6]. It is the leading cause of eosinophilic meningitis in humans worldwide and has affected >2800 people in at least 30 countries since its discovery as an agent of disease in 1945 [7,8]. In the definitive hosts (*Rattus* spp.) the larvae travel to the central nervous system (CNS) before maturing and reproducing in the pulmonary arteries [9]. This neural larval migrans also occurs in accidental hosts, which in synergy with an intense host immune reaction is responsible for neurological disease. The hallmark sign of NA is eosinophilic meningitis [10]. Definitive, accidental and paratenic hosts are infected with *A. cantonensis* following ingestion of infective 3^rd^ stage larvae (L3s), which develop inside the intermediate gastropod host from 1^st^ stage larvae (L1s) initially shed in infected rat faeces [11].

Among the many accidental hosts, primates appear to suffer from severe disease sequelae. Globally, including in Australia, multiple cases of captive non-human primate NA have been reported, which has obvious associated welfare concerns in zoological settings [[12], [13], [14], [15], [16], [17]]. Difficulties in controlling contact between definitive and intermediate hosts and captive primates in zoos are complicated by the need for environmental enrichment provided through fresh food (which attracts rats and gastropods) and the propensity for some small primate species to consume slugs and snails [18].

*Angiostrongylus cantonensis* has been established along Australia's east coast for at least 50 years, with the first case of human NA being documented in Brisbane [19,20]. NA in canines is regularly reported in Sydney and Brisbane, and many species of native Australian animals - such as macropods, possums, birds, and fruit bats - are known to be occasional accidental hosts [[21], [22], [23], [24], [25], [26], [27]]. The establishment and maintenance of *A. cantonensis* in eastern Australia is attributable to the successful invasion of rats (*R. rattus* and *R. norvegicus*); and various molluscan hosts, which potentially carry *A. cantonensis* infective stages [9,[28], [29], [30], [31]]. Two haplotypes of *A. cantonensis* have been identified in Australia via partial *cox*1 sequencing; the local endemic SYD.1, and the invasive Ac13 [[32], [33], [34]]. The distribution and pathogenesis of these two haplotypes is yet to be determined. A closely related species, *Angiostrongylus mackerrasae*, is native to Australia and possesses an identical life cycle and includes neural migrans in the definitive host – which is predominantly native rat species *R. fuscipes* and *R. luterolus* [9,35,36]. *A. mackerrasae* has never been implicated in human NA cases however, patent infection was described in a flying fox [37]. There is speculation that infection with *A. mackerrasae* might have gone unrecognised due to assumptive diagnosis [38]; although an effort has been made recently to genetically speciate the causative *Angiostrongylus* in canine cerebrospinal fluid (CSF) using ITS-2 sequencing [32,39].

In 2022, an 11-year-old male Bolivian squirrel monkey (*Saimiri boliviensis boliviensis*) at Sydney Zoo, New South Wales (NSW) Australia, developed a case of severe neural angiostrongyliasis and required euthanasia given the animal's grave prognosis. This case motivated an epidemiological investigation. We aimed to characterise and compare whole mitochondrial (mt)DNA of the *Angiostrongylus* nematode retrieved from the squirrel monkey to other Australian specimens to confirm its origin. We sought to screen the distribution of *Angiostrongylus* in the zoo rat population, and genetically confirm *Angiostrongylus* species and haplotype(s) via partial *cox*1 sequencing and rule out any co-infections with *A. mackerrasae* using ITS-2 next-generation sequencing (NGS) metabarcoding. To investigate the involvement of intermediate hosts in this case, we aimed to collect terrestrial gastropods for *Angiostrongylus* detection via hypersensitive quantitative (q)PCR. Due to concerns of an outbreak in other housed primates, we aimed to determine the extent of the zoo's *Angiostrongylus* problem and identify any high-risk clusters using an equal sampling effort survey targeting rat faeces.

## 2 Materials and methods

### 2.1 Ethics statement

Samples collected for the purposes of this research included the squirrel monkey's brain material, trapped brown rats (*Rattus norvegicus*), rat faeces and terrestrial gastropods. Mammalian tissue samples were collected from animals which were already dead; and were not killed for research/teaching purposes. A notification of tissue sample use (for squirrel monkey brain material and rat carcasses) was approved by Sydney University's Animal Ethics Committee (project number 2022/2242). The squirrel monkey was euthanised by a registered veterinarian on medical grounds, having a grave prognosis for recovery despite intensive care. Invasive brown rats were trapped and euthanised on private property according to Sydney Zoo's pest control measures. Gastropods collected excluded the two species listed on local conservation acts (*Pommerhelix duralensis* on the EPBC Act and *Meridolum corneovirens* on the NSW Biodiversity Conservation Act).

### 2.2 The Bolivian squirrel monkey (*Saimiri boliviensis boliviensis*)

#### 2.2.1 Necropsy and brain histology

Haematoxylin and eosin (H&E) stained slides of the brain and brainstem were examined by Vetnostics. Gross examination of the brain revealed a single nematode on the surface of the brain. A wet mount of the specimen was prepared and examined under a light microscope (Olympus BX41, Australia) at 40-400× magnification. The species of nematode was determined according to descriptions in Valentyne et al. [36] and Bhaibulaya [35].

#### 2.2.2 Species and haplotype (*cox*1) identification of the squirrel monkey's worm

DNA was purified from a medial segment of the nematode with the Monarch® Genomic DNA Purification Kit (New England Biolabs, Australia). DNA was stored at −20 °C. A quantitative (q)PCR assay targeting a 250 bp partial *cox*1 sequence using forward (AngiCOI_forward [S0963]) and reverse (AC1R [S0966]) as previously described [32]. The targeted region includes 16 single nucleotide polymorphisms (SNPs) which differ between *A. cantonensis* (MK570631.1) and *A. mackerrasae* (NC_046586); and also demarcates three SNPs at positions 159, 162, and 213, which discriminate the two known haplotypes present in Australia – the invasive Ac13 (KU532146.1) and local SYD.1 (MK570631.1) [32]. The amplicon was bidirectionally sequenced at Macrogen Inc. (Seoul, Korea) and chromatograph analysed in CLC Main Workbench v22 (Qiagen, CLC bio).

#### 2.2.3 mtDNA comparison of the squirrel monkey's worm (VM1) to two specimens sourced from rats

An individual male *A. cantonensis* specimen (R1M1) sourced from a brown rat (*R. norvegicus*) at Sydney Zoo, and archived DNA from a male *A. cantonensis* specimen (P48/19-B, CSIRO N5733) sourced from a black rat (*R. rattus*) in St Lucia, QLD in 2014 [in 36] were selected for comparative mtDNA analysis. DNA was isolated as described above. DNA from VM1, R1M1 and P48/19-B were submitted for whole genome sequencing using the Illumina NovaSeq 6000 platform (paired-end 150 bp reads; target depth of 1 GB raw data; Novogene AIT Genomics, Singapore). Consensus mtDNA sequences (mitogenomes) for VM1, R1M1 and P48/19-B were assembled from FastQ files using the GetOrganelle tool in Anaconda3 [40] via the University of Sydney's High Performance Computing system, Artemis (Sydney Bioinformatics Hub). Complete mtDNA nucleotide sequences for VM1, R1M1, P48/19-B and SYD.1 (MK570631.1) were aligned in CLC Main Workbench v22 (Qiagen, CLC bio) and a pairwise nucleotide difference table was created with identity (%) and number of differences (Supplementary Table S1). A maximum likelihood phylogenetic analysis tree was constructed in MEGA11 [41]. To inspect read coverage at a conflict between VM1 and R1M1 in the 2866–2875 bp region, FastQ files were trimmed for using the TrimGalore! [42] in Galaxy Australia [43], assembled with Bowtie2 [44] and mapped onto the VM1 mtDNA sequence for visualisation in IGV (Integrative Genomics Viewer [45]).

### 2.3 *Angiostrongylus* in trapped rats (*Rattus norvegicus*) and opportunistically collected rat faeces

#### 2.3.1 Sample collection and processing

Trapped rats (*n =* 14) were collected during September–October 2022 and euthanised on-site by a registered veterinarian according to Sydney Zoo's pest control protocols and immediately frozen. Carcasses were transported to the University of Sydney. Rats were identified, thawed, and the cardio-pulmonary system was meticulously searched for adult and sub-adult nematodes. All extracted worms were counted, rinsed with 0.9% phosphate buffered saline (PBS) and preserved in 80% ethanol at 4 °C. Species was confirmed for each of the *Angiostrongylus* specimens obtained via necropsy using morphological techniques outlined in 2.2.1. One male, and one female specimen from each rat were randomly selected for *cox*1 haplotyping, as described in 2.2.2. Faecal samples were collected from the rectum, or from the descending colon if rectal faeces was not available, and stored at 4 °C until further processing.

Rat faecal samples were opportunistically collected over two weeks in September 2022 from zoo back-of-house (BOH) and exhibit areas where rat activity had been observed by zoo staff. Faecal pellets were pooled together weekly for each site, and mixed by maceration and re-hydration with deionised water (ddH_2_O) where necessary. Pooled samples were stored at 4 °C until further processing.

#### 2.3.2 *Angiostrongylus* molecular identification and quantification

To identify *Angiostrongylus* species and *cox*1 haplotype, partial *cox*1 was amplified from ITS-2-positive samples (see section 2.2.2). Successfully amplified products (i.e. Ct < 35, and melt curve profiles matching positive control) were submitted for Sanger sequencing at Macrogen Inc. (Seoul, Korea).

To estimate the faecal load of *Angiostrongylus* L1s in trapped rat faeces and pooled opportunistically collected rat faecal samples, we used a probe-based qPCR assay targeting a 130 bp region of *Angiostrongylus* ITS-2 [46]. DNA was isolated from approximately 100 mg of each faecal sample using the ISOLATE II Fecal DNA Kit (Bioline, Australia); with 40 s of lysis in the FastPrep-24 (speed setting 6.0) bench-top bead beating lysis system (MP Biomedicals, Australia), and eluted at a final volume of 80 μl. Five 10-fold dilutions of DNA from known quantities of *A. cantonensis* L1s were included in each run to develop a standard curve for absolute quantification (efficiency (E) > 99%, and R^2^ ≥ 95). Reactions were run at a final volume of 20 μl, including 10 μl of Luna® Universal qPCR Mastermix (New England Biolabs, Australia) in a BioRad CFX96 Touch Real-Time PCR Detection System (BioRad, Australia). Thresholds were auto-calculated, and samples were considered positive if Ct < 35. To ensure the qPCR was able to detect positive faeces in pooled samples with high quantities of *Angiostrongylus*-negative faeces, mixes of *Angiostrongylus*-positive and -negative faeces sourced from Wistar rats at Westmead (Western Sydney Local Health District (WSLHD) Animal Ethics Committee approval number: 8003.03.18) were prepared at ratios of 1, 1:1, 1:10 and 1:100, isolated and amplified as above.

#### 2.3.3 Faecal ITS-2 next-generation sequencing (NGS) to confirm *Angiostrongylus* species

We adopted the ITS-2 qPCR assay (see 2.3.2) for amplicon metabarcoding and Next-Generation Sequencing (NGS) to allow us to rule out any undetected co-infections with *A. mackerrasae*. The target amplicon includes a G/A SNP at the 83^rd^ position which discriminates *A. cantonensis* from *A. mackerrasae* [32]. Reactions were run at a final volume of 30 μl, including 15 μl SensiFAST SYBR Probe No-ROX Mix (Bioline, Australia), 2 μl faecal DNA, and forward (S1060) and reverse (S1061) primers at a final concentration of 0.33 nM each. Cycling conditions were as follows: 95 °C for 3 min, then 40 cycles of 95 °C for 5 s, 60 °C for 10 s, and 72 °C for 20 s; with a final extension at 95 °C for 10 s and melt-curve analysis. Samples which were successfully amplified (Ct < 35) and produced melt-curve profiles matching positive controls were subjected to amplification and processing for NGS at the Ramaciotti Centre for Genomics, University of New South Wales, Australia using Illumina MiSeq v2 250 PE. Obtained FastQ files were processed using a local DADA2 pipeline [47] to create amplicon sequence variant (ASV) counts per sample. ASVs matching *Angiostrongylus* were visually verified in CLC Main Workbench v22 (Qiagen, CLC bio). Low coverage ASVs (< 3%) were discarded as spurious sequences. To validate the above NGS workflow to detect *A. mackerrasae* and *A. cantonensis* in mixed samples, additional duplicate reactions using neat and mixed archived *A. cantonensis* and *A. mackerrasae* DNA (at ratios 1:1, 1:10, 1:100, 100:1, 10:1) were processed in the same manner.

### 2.4 Collection of terrestrial gastropods and detection of *Angiostrongylus* via hypersensitive qPCR assay

Throughout September–November 2022, zoo staff collected any gastropods found on zoo property which were then frozen at −4 °C. DNA from ∼25 mg of gastropod tissue cut from the foot was isolated using the Monarch® Genomic DNA Purification Kit (New England Biolabs, Australia). *Angiostrongylus* DNA was detected using a hypersensitive qPCR assay (AcanR3990) [48] with reagents and cycling conditions as described in Baláž et al. [39]. Reactions were performed in duplicate and samples were considered positive if both Ct-values were ≤ 40, suspect if only one replicate achieved a Ct-value of ≤40, and negative if there was no amplification in either replicate.

### 2.5 Mapping *Angiostrongylus* risk using rat faeces as a proxy

#### 2.5.1 Equal sampling effort (ESE) faecal collection and *Angiostrongylus* detection

In an effort to ensure equal sampling effort across the zoo property, zookeepers from 29 back-of-house (BOH) areas were recruited for rat faecal sample collection. Each week for 4 consecutive weeks (from 10^th^ October – 6^th^ November 2022) zoo staff collected rat faecal samples found during daily cleaning of BOH areas. The number of faecal pellets collected per week was recorded. Pooled weekly samples (referred to as “ESE” samples from hereon) were mixed and DNA isolated as previously described. The ITS-2 qPCR assay (see 2.3.3) was utilised on the pooled samples for *Angiostrongylus* detection and L1 quantification, along with positive and blank extraction controls. Samples with amplification of Ct < 35 were considered positive.

#### 2.5.2 Cluster analyses

Each of the back of house (BOH) sites included in the survey (*n =* 29) were assigned a cartesian (x, y) coordinate, according to their location on the property. The number of rat faecal pellets collected per week, per site, were summed. To identify any clustering of successful rat faces collection, this data were analysed using a retrospective space-time permutation model. To investigate clustering of positive *Angiostrongylus* detections, data points (1 for positive, 0 for negative) were scanned using a retrospective space-time analysis for high proportion clusters (Bernoulli model). Both models were run using SaTScan v9.6 [49].

## 3 Results

### 3.1 The culprit in the squirrel monkey's case was *Angiostrongylus cantonensis*, haplotype Ac13

#### 3.1.1 Clinical history

**Fig. 1:**
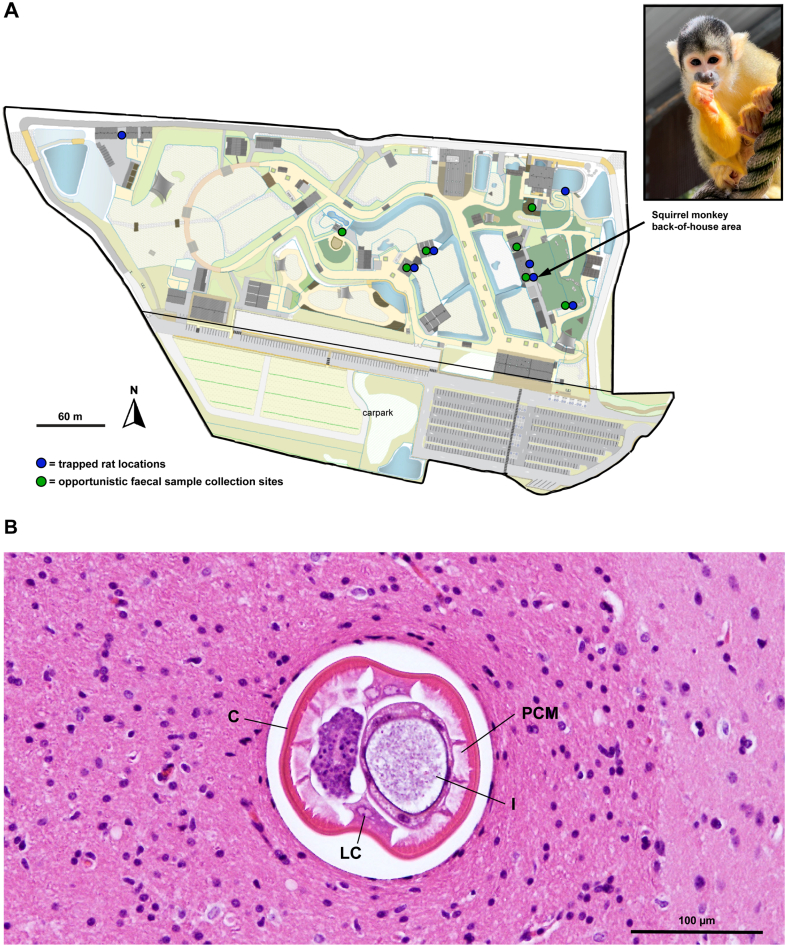
Neural angiostrongyliasis case in a Bolivian squirrel monkey at Sydney Zoo, NSW, Australia. (A) Map of Sydney Zoo displaying sites where trapped brown rats (*Rattus norvegicus*) and rat faeces were opportunistically collected for *Angiostrongylus* investigation. The location where the Bolivian squirrel monkey (*Saimiri boliviensis boliviensis*), was housed when he succumbed to neural angiostrongyliasis is indicated. Photo credit: Ellen Marfleet, Sydney Zoo. (B) Haematoxylin and eosin (H&E) stained slide of tissue taken from the brainstem of the squirrel monkey. The cross-section of a nematode with morphology consistent with *Angiostrongylus cantonensis* is apparent. LC = lateral cord, PCM = polymyarian-coelomyarian musculature, I = intestine, C = cuticle. (For interpretation of the references to colour in this figure legend, the reader is referred to the web version of this article.)

Vivo, an 11-year-old, male, Bolivian squirrel monkey (*Saimiri boliviensis boliviensis*) at Sydney Zoo, NSW Australia, developed a case of severe neural angiostrongyliasis (Fig. 1A). On the 31^st^ of May 2022, five months after translocation to the zoo, the squirrel monkey presented with sudden onset ataxia and lethargy. Oral meloxicam and paracetamol was prescribed, but he soon progressed to having hindlimb paresis. The squirrel monkey was then taken to a veterinary referral centre for further diagnostics which included: full blood count, standard biochemistry, faecal screening for internal parasites, bacterial culture, serology for toxoplasmosis as well as a computed tomography (CT) scan and magnetic resonance imaging (MRI). Results were inconclusive and through a diagnosis of exclusion there was a high suspicion for neural angiostrongyliasis. Cerebrospinal fluid collection was not attempted due to his small size. He was subsequently prescribed systemic steroids, anthelmintics, broad-spectrum antibiotics and given supportive care. On the 12^th^ of June 2022, the decision was made for compassionate euthanasia due to the poor prognosis for return to normal function and associated welfare implications. A complete post-mortem examination was conducted at the zoo and samples collected for histopathology.

After discussion with staff, it became apparent that the property has a large population of pestiferous rats which exploit resources such as fresh fruit and vegetables available on exhibit. They also reported that there was the occasional appearance of slugs near the back of house area of the exhibit. This individual monkey was known to be a curious forager and would often be seen on the ground looking for insects to eat, where he may have encountered a slug.

#### 3.1.2 Histology

Histological H&E slides prepared from the squirrel monkey's cerebrum, brain stem and spinal cord revealed multifocal small regions of haemorrhage and malacia surrounded by variable numbers of macrophages. Additionally, small numbers of mixed inflammatory cells comprising eosinophils, plasma cells and macrophages were present within the meninges. Within the brainstem was a cross section of a nematode, *150* μm diameter, containing a large intestine with few multinucleated cells, lateral chords and polymyarian-coelomyarian musculature (Fig. 1B). The morphology of the nematode is consistent with descriptions of *Angiostrongylus*. A similar nematode was present in the cerebrum. At the base of the brainstem, the meninges contained a degenerate nematode associated with mineralisation, granulomatous inflammation and multinucleate giant cells.

#### 3.1.3 Morphology

**Fig. 2:**
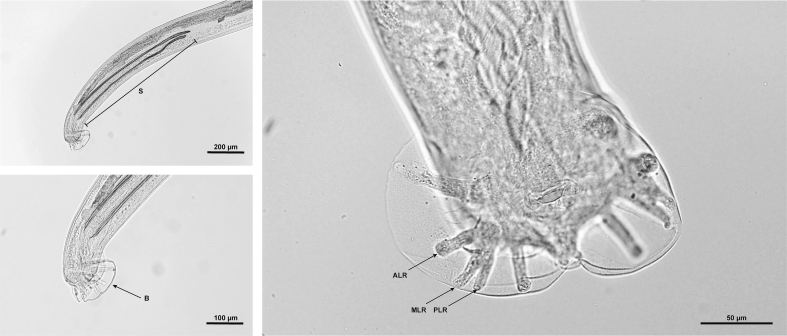
Wet mount of an immature male *Angiostrongylus cantonensis* specimen found on the surface of the brain of a Bolivian squirrel monkey (*Saimiri boliviensis boliviensis*) with neural angiostrongyliasis. S = spicule, B = bursa, ALR = antero-lateral ray, MLR = medio-lateral ray, PLR = postero-lateral ray.

The whole nematode collected from the brain was determined to be a sub-adult *Angiostrongylus cantonensis* male specimen, as per morphological descriptions in Valentyne et al. [36] and Bhaibulaya [35]. Distinctive features of *A. cantonensis* which allowed for the differentiation of the specimen from the morphologically similar and native *A. mackerrasae* included the following: (1) spicules measuring 800–1300 μm, which is significantly longer than the average length of *A. mackerrasae* (421 μm), and (2) postero-lateral ray marginally shorter than the medio-lateral ray (Fig. 2).

#### 3.1.4 *Cox*1 haplotype

**Supplementary Fig. 1:**
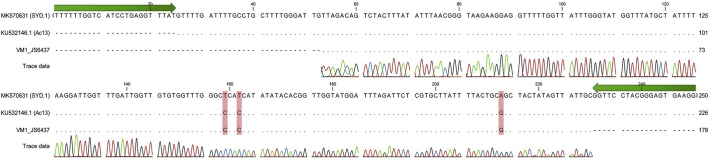
Alignment of a partial *cox*1 sequence (250 bp) obtained from the DNA of an immature male *Angiostrongylus cantonensis* specimen found on the surface of the brain of a Bolivian squirrel monkey (*Saimiri boliviensis boliviensis*) with neural angiostrongyliasis (VM1). The three single nucleotide polymorphisms (SNPs) which discriminate the two known haplotypes present in Australia, Ac13 (KU532146.1) and SYD.1 (MK570631), are highlighted red. Forward and reverse primers used (963, 966) for initial PCR amplification are shown in green.

Multiple sequence alignment of the trimmed partial *cox*1 sequence from the squirrel monkey's worm (VM1) revealed that VM1 shared partial *cox*1 genotype with *A. cantonensis* Ac13 (KU532146.1). Alignment of the VM1 sequence with downloaded sequences for *A. cantonensis* isolates Ac13 (KU532146.1) and SYD.1 (MK570631.1) reveals the three distinctive SNPs; C (cytosine) at the 159th position, C (cytosine) at the 162nd position and G (guanine) at the 213th position (Supplementary Fig. 1).

### 3.2 High conservation of mtDNA sequence between Ac13 specimens (VM1, R1M1 and P48/19-B)

**Fig. 3:**
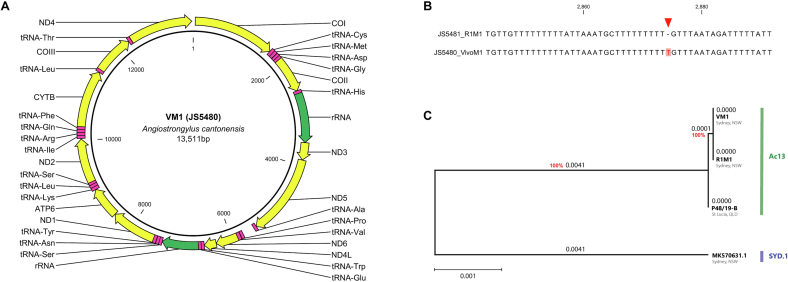
mtDNA analysis of *Angiostrongylus cantonensis* specimens. Consensus mtDNA sequence for VM1, R1M1 and P48/19-B was generated in this study by Illumina amplicon metabarcoding next generation sequencing and assembly via bioconda. (A) Circular mtDNA assembly and annotation of an *A. cantonensis* specimen sourced from a Bolivian squirrel monkey (*Saimiri boliviensis boliviensis*) with neural angiostrongyliasis at Sydney Zoo, NSW (VM1). (B) Alignment of complete mtDNA from VM1 and an *A. cantonensis* specimen sourced from a brown rat (*Rattus norvegicus*) from the same zoo property (R1M1). A single conflict (T deletion) within a poly-T region is flagged. (C) Phylogenetic tree comparing complete mtDNA nucleotide sequences obtained for VM1, R1M1, an archived *A. cantonensis* specimen from St Lucia, QLD (P48/19-B) and published SYD.1 (MK570631.1) inferred by using the Maximum Likelihood method and Tamura-Nei model. The tree is drawn to scale, with branch lengths measured in the number of substitutions per site (above the branches). 100 bootstrap replicates were run, and support values (%), shown in red were calculated. The tree was constructed in MEGA11: Molecular Evolutionary Genetics Analysis v11.0.13. The haplotype (determined via partial *cox*1 sequence) of each specimen is indicated – i.e. Ac13 or SYD.1. (For interpretation of the references to colour in this figure legend, the reader is referred to the web version of this article.)

Illumina NGS yielded a total of 9,297,216 (1.38 GB, 90.68% high quality Q30) raw reads for VM1, 10,655,672 (1.60 GB, 91.31% high quality Q30) raw reads for R1M1, and 3,134,806 (0.9 GB, 91.59% high quality Q30) raw reads for P48/19-B. The final assembled mtDNA sequences obtained from VM1, R1M1 and P48/19-B totalled lengths of 13,511 bp, 13,510 bp and 13,510 bp respectively. The trimmed partial *cox*1 sequences obtained by PCR (primers S0963 and S0966, 250 bp) from the same initial DNA following Sanger sequencing of VM1 and R1M1 were 100% identical to the mtDNA assembled from the NGS data, Upon analysis of the partial *cox*1 region, all three specimens matched the Ac13 haplotype. The circular mitogenome of VM1 was created and annotated (Fig. 3A). Alignment of the VM1 and R1M1 sequences in CLC Main Workbench allowed for the detection of a single conflict at the 2784–2875th position, where a T-deletion in the R1M1 sequence was observed in a ‘T' homopolymer region on an rRNA gene (Fig. 3B). Pairwise comparisons of identity (%) and number of nucleotide differences between the mitogenomes for VM1, R1M1, P48/19-B and SYD.1 (MK570631.1.1) ranged from 99.08 to 99.99% and 1–125, respectively (Supplementary Table S1**)**. Of the three Ac13 specimens (VM1, R1M1 and P48/19-B), there was very little variation in mtDNA sequence; only one T-to-G nucleotide substitution between R1M1 and P48/19-B (located on the tRNA-Arginine gene, position 10,141), and only two differences between VM1 and P48/19-B (T-deletion and T-to-G substitution located on rRNA and tRNA-Arginine, positions 2875 and 10,136, respectively). The Ac13 haplotypes had mtDNA that differed to the SYD.1 (MK570631.1) haplotype by 124–125 nucleotide bases (Supplementary Table S1). The maximum likelihood phylogeny tree comparing whole mtDNA from VM1, R1M1, P48/19-B and previously assembled SYD.1 (MK570631.1) isolates indicate extremely high conservation between VM1, R1M1 and P48/19-B, with high bootstrap support (100%) independent of where the specimen was initially collected (Fig. 3C).

### 3.3 *A. cantonensis* prevalence, shedding and *cox*1 haplotyping in trapped rats and opportunistically collected rat faeces

#### 3.3.1 64% of trapped rats were positive for *Angiostrongylus* at necropsy

**Supplementary Fig. 2:**
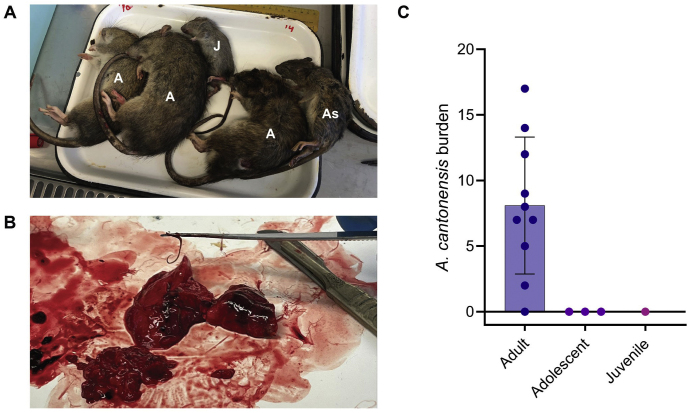
*Angiostrongylus* burden in the cardiopulmonary system of trapped brown rats (*Rattus norvegicus*) at Sydney Zoo, Bungarribee, NSW. (A) Five of 14 brown rats (R10-R14) from the current study, with life stage indicated in white; A = adult, As = adolescent, J = juvenile. (B) *Angiostrongylus* adult worms visible in the pulmonary arteries during necropsy of one brown rat (R2). (C) Scatterplot of total *Angiostrongylus cantonensis* burden of 14 trapped brown rats grouped according to life stage. Dots represent individual values, bars indicate the mean, and T-bars show the standard deviation.

Over the course of two months (September–October 2022) 14 rats (all identified as *Rattus norvegicus*) from 7 locations on the zoo property were trapped and euthanised according to Sydney Zoo's pest control protocols (Fig. 1A). Rats were identified as adults (*n* = 10), adolescents (*n* = 3) and juveniles (*n* = 1) (Supplementary Fig. 2A). Upon necropsy of the lungs and pulmonary arteries, 64% (9/14) were positive for *Angiostrongylus* nematodes (Supplementary Table S2**,** Supplementary Fig. 2B). All rats positive at necropsy were adults (Supplementary Table S2**,** Supplementary Fig. 2C). All nematodes (*n =* 81) matched morphological descriptions of *A. cantonensis*. Total worm counts (TWC) from *Angiostrongylus*-positive rats ranged from 2 to 17, with a mean of 9 and a male-to-female sex ratio of 0.62 (Supplementary Table S2).

#### 3.3.2 *Cox*1 haplotyping of adult worms shows co-infections with SYD.1 and Ac13

*Cox*1 haplotypes were successfully confirmed via Sanger sequencing and partial *cox*1 alignment for all randomly selected adult worm specimens (*n =* 18; 9 male, 9 female). Of the male specimens, 78% (7/9) were determined to match the Ac13 type, and 22% (2/9) matched the SYD.1 type. For the female specimens, 78% (7/9) were also determined to match the Ac13 type, and two (22%) matched the SYD.1 type. Two of nine rats (R5 & R10) were demonstrated to harbour co-infections with both Ac13 and SYD.1 haplotypes (Supplementary Table S2).

#### 3.3.3 Detection and quantification of L1s in rat faeces via ITS-2 qPCR

Seven out of nine (78%) trapped rats with *Angiostrongylus* found during necropsy, were positive via faecal ITS-2 qPCR (Supplementary Table S2). The remaining two rats positive at necropsy failed to amplify *Angiostrongylus* DNA (R1 & R5).

Over the two week period, 11 rat faecal samples from 7 sites were opportunistically collected and pooled together (Fig. 1A). The majority (9/11; 82%) were positive via ITS-2 qPCR. The average SQ (starting quantity) for all positive trapped rats and opportunistically collected faecal samples was 1074.4 L1s/100 mg, ranging from 1.5 to 3090 L1s/100 mg (Supplementary Table S2). Validation of the ITS-2 qPCR assay's sensitivity revealed that it was able to detect *Angiostrongylus* in mixed samples with as little as 1/100th of *Angiostrongylus*-positive faeces mixed with uninfected rat faeces.

#### 3.3.4 *Cox*1 haplotyping of opportunistically collected rat faeces

Of the nine ITS-2 qPCR-positive pooled opportunistically collected rat faecal samples, eight had their 250 bp partial *cox*1 region successfully sequenced, and one (SMB-1) failed. After trimming for quality, multiple sequence alignment revealed that 88% (7/8) possessed the 3 SNPs that are unique to the Ac13 haplotype. One sample (BB-2) matched the SYD.1 *cox*1 haplotype.

#### 3.3.5 Absence of coinfection with *A. mackerrasae* via faecal ITS-2 NGS

**Supplementary Fig. 3:**
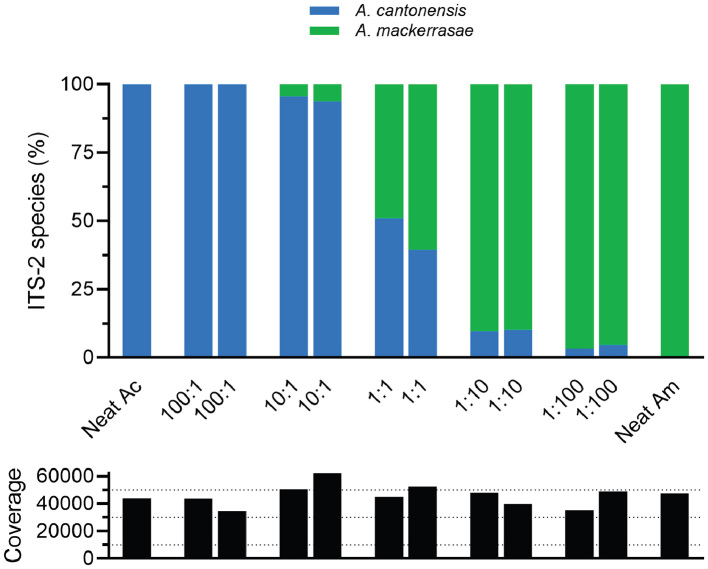
Proportion of *A. cantonensis* (Ac) and *A. mackerrasae* (Am) DNA in a mock-community of mixed samples identified via partial ITS-2 Next-Generation Sequencing (NGS) and DADA2 analysis. Initial amplification PCRs included archived neat *A. cantonensis* and *A. mackerrasae* DNA and samples mixed at ratios of 1:1, 1:10, 1:100, 100:1, 10:1. Coverage is displayed as total number of reads per sample.

A total of 1,162,719 raw reads in FastQ format from 16 faecal samples and 12 validation samples were produced via Illumina NGS and processed through the DADA2 pipeline. After filtering, denoising, merging forward and reverse reads, and chimera removal, 1,143,199 reads remained for analysis. A total of 24 ITS-2 Amplicon Sequence Variants (ASVs) were produced, four of which possessed the *A. mackerrasae* SNP, and the remaining 20 were *A. cantonensis*. After excluding values with <3% reads, proportions of *A. cantonensis* and *A. mackerrasae* reads per sample were calculated. All trapped rat (*n =* 7) and opportunistically collected faecal samples (*n =* 9) were identified to contain only *A. cantonensis* ASVs. Validation ITS-2 NGS samples (varying ratios of mixed DNA from *A. cantonensis* and *A. mackerrasae*) processed in the same manner accurately detected *Angiostrongylus* species proportions in mixed samples which contained ≥10% of *A. mackerrasae* species DNA (Supplementary Fig. 3).

### 3.4 Rat faeces were found primarily in the primate and herbivore exhibits, and there was no evidence of *Angiostrongylus*-positive faeces clustering across the zoo

**Fig. 4:**
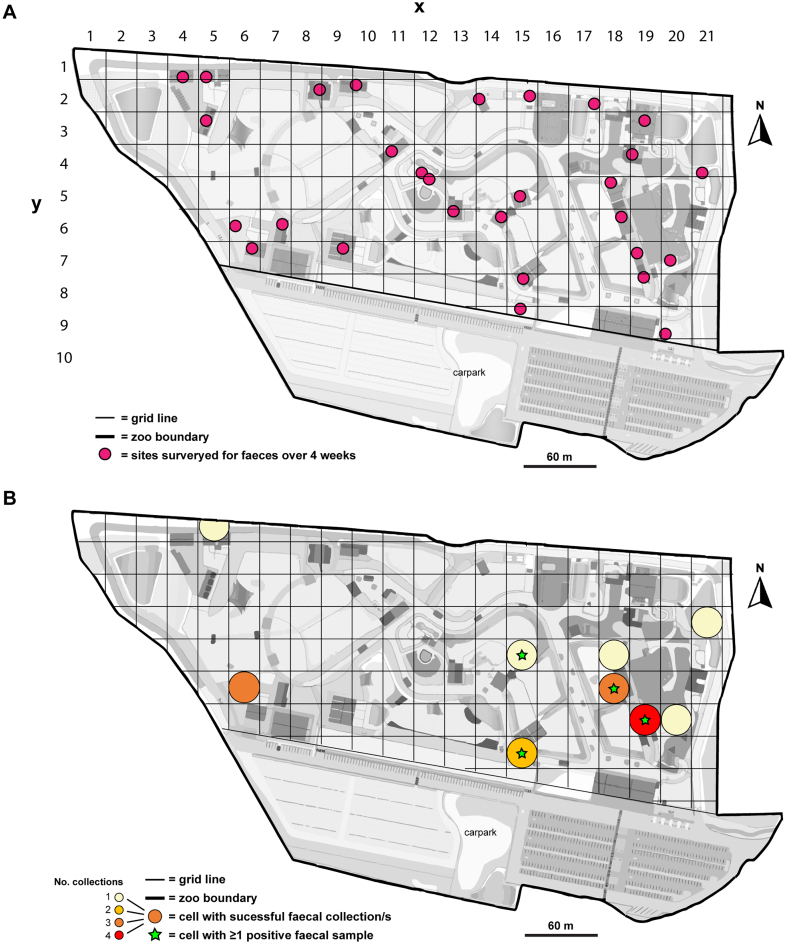
Mapping equal sampling effort (ESE) faecal samples collected at Sydney Zoo (western Sydney, NSW, Australia) for the current study. (A) Map displaying the 29 back-of-house (BOH) sites (with cartesian (x, y) coordinates), which were recruited for a zoo-wide survey for rat faeces (=rat activity) and *Angiostrongylus* detection. Each week, for four consecutive weeks (from 10th October – 6th November 2022), zoo staff pooled together rat faecal samples found during daily cleaning of BOH areas. A qPCR targeting the ITS-2 region of *Angiostrongylus* spp. was performed on the pooled samples for *Angiostrongylus* detection and L1 quantification. (B) Results from ESE rat faeces collection and molecular detection of *Angiostrongylus*. Cells where successful collection/s of rat faeces were made are indicated with circles of varying colour, depending on the number of collections made over 4 weeks. Cells where pooled rat faeces was positive for *Angiostrongylus* at least once over the four-week period, are marked with a star.

Twenty-nine back of house (BOH) areas were recruited for rat faecal sample collection and assigned cartesian (x, y) coordinates according to their relative location (Fig. 4A**,** Table 1). Over the four weeks, a total of 18 pooled equal sampling effort (ESE) faecal samples from nine sites (Australiana (x = 18, y = 5), baboon (x = 18, y = 6), capuchin (x = 15, y = 5), chimpanzee (x = 15, y = 8), giraffe (x = 5, y = 1), nyala (x = 6, y = 6), squirrel monkey (x = 19, y = 7), tammar wallaby (x = 21, y = 4) and wombat (x = 20, y = 7)) were obtained (Fig. 4B**,** Table 1). Only two sites (baboon and squirrel monkey) had faecal samples successfully collected every week for four weeks. Eight samples from four sites (baboon, capuchin, chimpanzee and squirrel monkey) were ITS-2 qPCR-positive for *Angiostrongylus* (Fig. 4B). The average SQ for PCR-positive ESE samples was 207.7 L1s/100 mg, ranging from 0.6 to 395.5 L1s/100 mg (Table 1).

**Table 1:** Location and *Angiostrongylus* detection data for rat faeces successfully collected across Sydney Zoo with equal sampling effort.

Faecal collections			Location		Faecal ITS-2 qPCR		
ID	Week	No. faecal pellets	Site	x, y	Ct-value	SQ	PCR call*
*E*-A4	4	12	Australiana	18, 5	N/A	N/A	NEGATIVE
E-B1	1	21	Baboon	18, 6	21.2	289.8	POSITIVE
E-B2	2	12			21.1	321.4	POSITIVE
E-B3	3	11			N/A	N/A	NEGATIVE
E-B4	4	4			N/A	N/A	NEGATIVE
E-C1	1	11	Capuchin	15, 5	22.5	113.3	POSITIVE
E-CH1	1	6	Chimpanzee	15, 8	N/A	N/A	NEGATIVE
E-CH2	2	7			23.9	40.2	POSITIVE
E-G3	3	7	Giraffe	5, 1	N/A	N/A	NEGATIVE
E-N1	1	6	Nyala	6, 6	N/A	N/A	NEGATIVE
E-N3	3	13			N/A	N/A	NEGATIVE
E-N4	4	14			N/A	N/A	NEGATIVE
E-SMB1	1	28	Squirrel Monkey	19, 7	29.7	0.6	POSITIVE
E-SMB2	2	40			20.8	395.5	POSITIVE
E-SMB3	3	9			20.9	364.9	POSITIVE
E-SMB4	4	25			22.3	136.2	POSITIVE
E-TW1	1	1	Tammar Wallaby	21, 4	N/A	N/A	NEGATIVE
E-W1	1	8	Wombat	20, 7	N/A	N/A	NEGATIVE

Ct = cycle threshold, SQ = starting quantity (L1s/100 mg), N/A = null Ct-value (i.e. no amplification). *PCR call was positive if Ct < 35.

Scanning cluster analysis for successful faecal sample collections detected five statistically significant (*p* < 0.05) clusters – one primary, and four secondary clusters. The primary cluster included four sites, zebra (x = 5, y = 3), giraffe (x = 5, y = 1), nutrition (x = 4, y = 1) and nyala (x = 6, y = 6) during week 3. Secondary clusters were Australiana (x = 18, y = 5) during week 4; capuchin (x = 15, y = 5) during week 1; squirrel monkey (x = 19, y = 7) during week 2; and wombat (x = 20, y = 7) during week 1. Scanning for clusters with high rates of *Angiostrongylus* detections in collected faeces (using the Bernoulli model consisting of cases and controls) identified only one cluster, although it was not statistically significant (*p* = 0.173).

### 3.5 Collected slugs were negative upon hypersensitive *Angiostrongylus* qPCR assay

During the entire course of the study (September–November 2022) study, only 3 gastropods were found and collected. One gastropod was visually identified as a leopard slug (*Limax maximus*). The remaining two small (< 150 mm) slugs were unable to be identified due to the freeze-thaw process damaging distinguishing features. DNA from all gastropod specimens gave a negative (N/A Ct-value) result using AcanR3990 qPCR.

## 4 Discussion

Here we detailed a case and epidemiology of neural angiostrongyliasis (NA) in a captive Bolivian squirrel monkey (*Saimiri boliviensis boliviensis*) in Australia caused by the invasive parasite *A. cantonensis* (rat lungworm). The clinical findings for the squirrel monkey were consistent with typical presentations found in non-human primate NA cases, including lethargy, ataxia, dysstasia and peripheral paresis [[12], [13], [14], [15], [16], [17]]. Histologically, the infiltration of eosinophils and macrophages in the meninges accompanied with haemorrhage is consistent with pathology secondary to intracranial nematode infection. As with a majority of *A. cantonensis* infections in aberrant accidental hosts, the nematodes found in the CNS were in a sub-adult stage [26,50].

The case led to an epidemiological investigation to attempt to identify the level of exposure and prevalence of infection in the resident rat population within the zoo and potentially identifying clusters of infected rat faeces that could lead to improvements in risk-mitigation activities for *A. cantonensis* infection. First, we devised an equal sampling effort (ESE) approach for cluster analysis that indicates an increased risk and significant clustering of rat faeces predominantly around zoo exhibits with availability of fresh food provided to zoo animals and lack of predatory animals, such as large felids. Analysis of *Angiostrongylus*-positive faecal samples failed to detect significant clustering, implying that the distribution of *Angiostrongylus* in rat faeces during the short study period was uniform across the zoo property. As shedding of L1s by infected rats is a widespread issue, any species of slug or snail in this area are at high risk to become an infected intermediate host. Susceptible accidental hosts on exhibit (marsupials and primates), visitors (a curious toddler, for example) and wildlife that might enter the zoo (e.g. tawny frogmouths, possums, flying foxes) are therefore at risk of infection [14,21,22,26,37,51]. An extensive rat control program that involves baiting, trapping and shooting is already in place at the zoo. Proximity of the zoo to the nature reserve will make the control of *A. cantonensis* infection and rat populations difficult, as it is likely that the majority of rats outside the zoo will be *A. cantonensis* positive as well, serving as the reservoir for the zoo population. Following the study, zoo staff were able to allocate budget for the implementation of more focussed control measures (such as filling in burrows, rodent culls, and reducing leftover food in exhibits) and rodent-proofing in higher risk areas. Since then, staff have reported a decrease in rat activity. Although unconventional, future zoo design could exploit the rat's avoidance of areas where predators are exhibited, and place at-risk species within low rat activity areas to reduce cross over of infected rat faeces and susceptible zoo animals - however, this solution may introduce other welfare and access issues. Slugs - including exotic leopard slugs (*Limax maximus*) - have been observed across the zoo property, although in extremely small numbers. *L. maximus* is known to be a suitable host for *A. cantonensis*, with natural infections of >300,000 larvae per individual previously estimated [31]. Aware of this, Sydney Zoo had already implemented an exhaustive pest-control regime including the placement of beer-traps, Perspex barriers and electric lines, in addition to hygienic feeding and cleaning practices. This mitigation activity likely contributed to only three slugs collected during the intensive survey of the zoo that targeted both the rat faeces as well as gastropods (slugs and snails). Due to the small sample size, we cannot comment on the prevalence of *Angiostrongylus* in intermediate hosts, however the negative PCR results for all three specimens suggests *Angiostrongylus* outbreaks may be extremely localised and contingent on the abundance of gastropods. A similar study extending for a longer period of time targeting likely paratenic hosts would be useful for further describing *Angiostrongylus* epidemiology in such a context.

Morphology and *cox*1 sequence analysis of the worm found on the squirrel monkey's brain confirm that the culprit in this case was the invasive Ac13 haplotype of *A. cantonensis*. Presence of the Ac13 haplotype was recently confirmed to be present in dogs suffering from canine NA in Australia [32,39] together with the established SYD.1 haplotype [33]. Across the entire 13,511 bp mitogenome (mtDNA) of *A. cantonensis* from the squirrel monkey, only one nucleotide difference was observed when compared to a specimen extracted from a rat at the zoo property, and only two nucleotide differences were found when aligned with the mtDNA from an Ac13 specimen sourced from St Lucia, QLD (P48/19-B). A previously published mtDNA sequence of the SYD.1 haplotype originally sourced from Mosman in Sydney differed to VM1 and R1M1 by 124 and 125 nucleotides, respectively, after pairwise alignment. The extremely high conservation of the mitochondrial DNA within the *A. cantonensis* Ac13 group compared to the SYD.1 voucher specimen confirms their historical genetic divergence and clear establishment of separate lineages in Australia, possibly due to separated introduction events [32].

Naturally acquired *A. cantonensis* infections in non-human accidental hosts have been associated with increased intermediate host activity, seen after warm wet weather in autumn (which occurs March–May in Australia [52]). A seasonal peak in canine NA cases during May was observed in Sydney by Walker et al. [53], which coincides with (1) higher abundance of terrestrial gastropods, and (2) the 9–14 day prepatent period for canine hosts [24,54]. The prepatent period for human cases ranges from 1 day to several months (average 11 days [6]), which is consistent with experimental infections in some non-human primates [55,56]. The appearance of clinical signs in the squirrel monkey began in May 2022 (euthanasia 12 days later), mirroring these seasonal aspects of canine NA. It was likely that the squirrel monkey acquired infection via consumption of an infected intermediate or paratenic host, as he was known for his curiosity and foraging; however, the potential for infective larvae to have been released by a mollusc into his drinking water supply should also be considered [[57], [58], [59]].

A handful of surveys have partially described the distribution of *Angiostrongylus* spp. infection in invasive Australian rats. Trapping and post-mortem of *R. rattus* on the Beecroft Peninsula, NSW (200 km south of Sydney) by Stokes et al. [60] estimated a presumed *A. cantonensis* prevalence of 4.4%, which peaked at 11.3% in summer. Higher prevalence was found in QLD by Aghazadeh et al. [29], where 16% *R. rattus* and 27% *R. norvegicus* were positive. Earlier surveys conducted in QLD demonstrated similar prevalence, ranging from 6.5 to 15.6% for *R. rattus* and 5–23% for *R. norvegicus* [28,35,61]. The “hotspot” of *A. cantonensis* at Sydney Zoo might be a consequence of the locality's urban-bushland interface; ideal for the parasite's proliferation [38,62]. The zoo is located in a large nature reserve, allowing rats to access food and defecate on zoo property and return to habitat where intermediate hosts are common, creating an overlap of definite, intermediate and accidental host ranges. Alternatively, the prevalence in brown rats might just be underreported or underestimated due to scant surveys of peri-urban rats in Sydney. Two rats in our study (R1 and R5) positive at necropsy failed to amplify *Angiostrongylus* DNA using the faecal ITS-2 qPCR, however R1 only harboured sub-adults and R5 had a total worm burden of two (1 male, 1 female), which is consistent with low/no shedding of L1s in faeces. Average total worm burden in the lungs of infected trapped brown rats was 9 (range 2–17), which is consistent with counts observed in previous surveys [29,60]. All rats that were positive for *A. cantonensis* at necropsy were fully grown, supporting the hypothesis that older rats are more likely to have been exposed to L3s, potentially multiple times [63].

We detected only *A. cantonensis* throughout this investigation in western Sydney, NSW using morphology, partial *cox1* sequencing and ITS-2 NGS metabarcoding. In Queensland, *R. norvegicus* are known to be co-infected with *A. cantonensis* and a native species of *Angiostrongylus* – *A. mackerrasae* [35]. The distribution of *A. mackerrasae* across eastern Australia is unknown and only Brisbane and Cairns in Queensland, and south of Jervis Bay in NSW are current known localities of this parasite [29,60]. Previously, Chan et al. [30] suggested the presence of *A. mackerrasae* in collected snails in Sydney, yet publicly available DNA sequences suggest that all their DNA belongs to *A. cantonensis* [36]. Our ITS-2 NGS metabarcoding was able to detect *A. mackerrasae* in an experimental mock-community, where co-infection of *A. cantonensis* and *A. mackerrasae* was identified accurately if at least 10% *A. mackerrasae* DNA was included. Lower thresholds of detection in mixed infections should be interpreted with caution due to inherent bleeding or hopping during Illumina sequencing [64].

Confirmation of *cox*1 *A. cantonensis* mtDNA haplotypes has not been explored in surveyed Australian rats until now. Although only 1 male and 1 female *Angiostrongylus* specimen per trapped rat underwent *cox*1 haplotype identification, the majority (78%, 14/18) of individual worms were determined to be the Ac13 haplotype. Additionally, the *cox*1 haplotype of positive pooled opportunistically collected faecal samples (*n* = 9) was successfully assigned for 8 samples, with 7/8 (88%) being confirmed as Ac13. The introduction of other haplotypes in Australia is unlikely as a previous study found that a majority of cases in dogs (9/10) were infected with the Ac13 haplotype of *A. cantonensis*, and only one with SYD.1 [32]. However, in other countries several other *cox*1 haplotypes of *A. cantonensis* are assumed to have independently invaded or evolved [[65], [66], [67]]. Co-infections with both Ac13 and SYD.1 haplotypes were detected via partial *cox*1 sequences of selected adult/subadult specimens in two rats in our study. Whether these *A. cantonensis* haplotypes can interbreed and/or cause different pathologies in natural or accidental hosts is unknown. A previous study from Brazil suggested that two different lineages (ac8 and ac9) have different biological profiles, with ac9 shedding a significantly larger number of L1 larvae at the beginning of the patent period in experimentally infected brown rats [68].

## 5 Conclusions

Here we comprehensively investigated a case of neural angiostrongyliasis (NA) in a captive Bolivian squirrel monkey at a zoo in Sydney, Australia. The study revealed that the control of *A. cantonensis* in rat populations will prove to be an ongoing issue, due to the zoo's proximity to a nature reserve; although risk-mapping allowed for more focused control measures to be implemented. An unconventional strategy involving placing susceptible species (such as primates and macropods) within a zone of rat-deterring predatory animals to minimise exposure to susceptible species was contemplated for future zoo designs. Existing intermediate host control measures, targeting gastropods, were found to be effective; although widespread shedding of L1s by infected rats make any gastropod on-site a potential source of infection for visitors, and captive and wild animals. We confirmed the high prevalence of the invasive *cox*1 haplotype (Ac13) of *A. cantonensis*, with mtDNA analysis showing high conservation within the Ac13 group. Co-infections with both Ac13 and SYD.1 *A. cantonensis* haplotypes were detected in rats, raising questions about pathogenesis and potential interbreeding between haplotypes. These findings emphasise the need for comprehensive control measures addressing both rat populations and intermediate hosts to mitigate the risk of *Angiostrongylus* infections in a zoo context.

The following are the supplementary data related to this article.

Supplementary Table S1 Complete mtDNA (bp) alignment percent (%) identity and number of nucleotide differences between four *A. cantonensis* isolates. Supplementary Table S1

Supplementary Table S2 Summary data for trapped rats and pooled opportunistically collected faecal samples. Supplementary Table S2

## Funding

This work was supported by the 10.13039/501100001774University of Sydney Research Training Program (RTP) Tuition Fee Offset, Sydney, NSW.

## CRediT authorship contribution statement

**Phoebe Rivory:** Conceptualization, Data curation, Formal analysis, Investigation, Methodology, Project administration, Validation, Visualization, Writing – original draft, Writing – review & editing. **Kresen Pillay:** Investigation, Methodology, Project administration, Resources, Writing – original draft, Writing – review & editing. **Rogan Lee:** Resources, Supervision, Writing – review & editing. **David Taylor:** Formal analysis, Writing – original draft, Writing – review & editing. **Michael P. Ward:** Formal analysis, Writing – review & editing. **Jan Šlapeta:** Conceptualization, Methodology, Project administration, Resources, Supervision, Writing – original draft, Writing – review & editing.

## Declaration of Competing Interest

The authors have none to declare.

## Data Availability

Supplementary material and data generated for this study is available on LabArchives at https://dx.doi.org/10.25833/484s-bg84. Original FastQ files generated by ITS-2 Next-Generation Sequencing and Whole-Genome Sequencing were submitted to the NCBI Sequence Read Archive (SRA) under the BioProject PRJNA983549; accession numbers SRR24959066–SRR24959093 and SRR24922452–SRR24922454, respectively. Assembled and annotated mitogenomes for the three A. cantonensis Ac13 specimens (VM1, R1M1 and P48/19-B) were deposited in GenBank under accession numbers OR177659-OR177661
