# Supplementary material for: Fatal neural angiostrongyliasis in the Bolivian squirrel monkey (Saimiri boliviensis boliviensis) leading to defining Angiostrongylus cantonensis risk map at a zoo in Australia
Source: One Health. 2023 Sep 15;17:100628. doi: 10.1016/j.onehlt.2023.100628 (PMC10665155; doi:10.1016/j.onehlt.2023.100628)
Supplement: Supplementary Table S1 — Complete mtDNA (bp) alignment percent (%) identity and number of nucleotide differences between four A. cantonensis isolates. [file mmc4.docx]

**Supplementary Table S1**. Complete mtDNA (bp) alignment percent (%) identity and number of nucleotide differences between four *A. cantonensis* isolates.

|  |  | Identity (%) | | | |
| --- | --- | --- | --- | --- | --- |
|  |  | VM1 | R1M1 | P48/19-B | MK570631.1 |
| No. differences | VM1 (Ac13) |  | 99.99 | 99.99 | 99.08 |
|  | R1M1 (Ac13) | 1 |  | 99.99 | 99.08 |
|  | P48/19-B (Ac13) | 2 | 1 |  | 99.08 |
|  | MK570631.1 (SYD.1)* | 124 | 125 | 124 |  |

*mtDNA sequence was published previously by Valentyne et al. [36].
Haplotype determined via partial *cox*1 sequence is indicated in brackets.
