# Supplementary material for: Fatal neural angiostrongyliasis in the Bolivian squirrel monkey (Saimiri boliviensis boliviensis) leading to defining Angiostrongylus cantonensis risk map at a zoo in Australia
Source: One Health. 2023 Sep 15;17:100628. doi: 10.1016/j.onehlt.2023.100628 (PMC10665155; doi:10.1016/j.onehlt.2023.100628)
Supplement: Supplementary Table S2 — Summary data for trapped rats and pooled opportunistically collected faecal samples. [file mmc5.docx]

**Supplementary Table S2**. Summary data for trapped rats and pooled opportunistically collected faecal samples.

| Specimen | | | Adult/subadult *Angiostrongylus* | | | | Faecal ITS-2 qPCR | | | Faecal *Angiostrongylus* identification | |
| --- | --- | --- | --- | --- | --- | --- | --- | --- | --- | --- | --- |
| **ID** | **Specimen** | **Location** | **TWC** | **Worm details** | **Morphology** | ***cox*1 haplotype ♂ / ♀** | **Ct-value** | **SQ** | **PCR call*** | **ITS-2 species (NGS)** | ***cox*1 haplotype** |
| R1 | Adult *R. norvegicus* ***♂*** | Nutrition | 17 | Subadults: 3 male, 14 female | *A. cantonensis* | Ac13 / Ac13 | N/A | N/A | NEGATIVE |  |  |
| R2 | Adult *R. norvegicus* ***♂*** | Nutrition | 9 | 3 male, 6 female | *A. cantonensis* | SYD.1 / SYD.1 | 17.6 | 3090.0 | POSITIVE | 100% *A. cantonensis* |  |
| R3 | Adolescent *R. norvegicus* ***♂*** | Orangutan | 0 |  |  |  | N/A | N/A | NEGATIVE |  |  |
| R4 | Adult *R. norvegicus* ***♂*** | Squirrel monkey | 0 |  |  |  | N/A | N/A | NEGATIVE |  |  |
| R5 | Adult *R. norvegicus* ***♀*** | Squirrel monkey | 2 | 1 male, 1 female | *A. cantonensis* | Ac13 / SYD.1 | N/A | N/A | NEGATIVE |  |  |
| R6 | Adult *R. norvegicus* ***♂*** | Orangutan | 7 | 4 male, 3 female | *A. cantonensis* | Ac13 / Ac13 | 21.2 | 290.3 | POSITIVE | 100% *A. cantonensis* |  |
| R7 | Adolescent *R. norvegicus* ***♂*** | Wombat | 0 |  |  |  | N/A | N/A | NEGATIVE |  |  |
| R8 | Adult *R. norvegicus* ***♂*** | Capuchin | 5 | 2 male, 3 female | *A. cantonensis* | Ac13 / Ac13 | 20.8 | 409.5 | POSITIVE | 100% *A. cantonensis* |  |
| R9 | Adult *R. norvegicus* ***♀*** | Capuchin | 12 | 6 male, 6 female | *A. cantonensis* | Ac13 / Ac13 | 19.8 | 853.1 | POSITIVE | 100% *A. cantonensis* |  |
| R10 | Adult *R. norvegicus* ***♂*** | Aquarium | 8 | 4 male, 4 female | *A. cantonensis* | SYD.1 / Ac13 | 18.9 | 1597.1 | POSITIVE | 100% *A. cantonensis* |  |
| R11 | Adult *R. norvegicus* ***♂*** | Aquarium | 14 | 4 males, 10 females | *A. cantonensis* | Ac13 / Ac13 | 18.1 | 3035.2 | POSITIVE | 100% *A. cantonensis* |  |
| R12 | Juvenile *R. norvegicus* ***♀*** | Squirrel monkey | 0 |  |  |  | N/A | N/A | NEGATIVE |  |  |
| R13 | Adult *R. norvegicus* ***♂*** | Squirrel monkey | 7 | 4 males, 3 females | *A. cantonensis* | Ac13 / Ac13 | 20.9 | 378.6 | POSITIVE | 100% *A. cantonensis* |  |
| R14 | Adolescent *R. norvegicus* ***♀*** | Squirrel monkey | 0 |  |  |  | 35.7 | 0.0 | NEGATIVE |  |  |
| AB-1 | Pooled faecal pellets (*n =* 3) | Australiana |  |  |  |  | 21.0 | 231.9 | POSITIVE | 100% *A. cantonensis* | Ac13 |
| AB-2 | Pooled faecal pellets (*n =* 5) |  |  |  |  |  | 23.4 | 75.1 | POSITIVE | 100% *A. cantonensis* | Ac13 |
| BB-1 | Pooled faecal pellets (*n =* 2) | Baboon |  |  |  |  | N/A | N/A | NEGATIVE |  |  |
| BB-2 | Pooled faecal pellets (*n =* 30) |  |  |  |  |  | 29.0 | 1.5 | POSITIVE | 100% *A. cantonensis* | SYD.1 |
| CB-2 | Pooled faecal pellets (*n =* 20) | Capuchin |  |  |  |  | 18.3 | 2399.1 | POSITIVE | 100% *A. cantonensis* | Ac13 |
| OB-1 | Pooled faecal pellets (*n =* 3) | Orangutan |  |  |  |  | N/A | N/A | NEGATIVE |  |  |
| OTB-2 | Pooled faecal pellets (*n =* 2) | Otter |  |  |  |  | 20.2 | 656.3 | POSITIVE | 100% *A. cantonensis* | Ac13 |
| SMB-1 | Pooled faecal pellets (*n =* 9) | Squirrel monkey |  |  |  |  | 21.1 | 209.5 | POSITIVE | 100% *A. cantonensis* | *Failed* |
| SMB-2 | Pooled faecal pellets (*n =* 20) |  |  |  |  |  | 19.6 | 994.6 | POSITIVE | 100% *A. cantonensis* | Ac13 |
| WB-2 | Pooled faecal pellets (*n =* 3) | Wombat |  |  |  |  | 18.6 | 1990.8 | POSITIVE | 100% *A. cantonensis* | Ac13 |
| WE-1 | Pooled faecal pellets (*n =* 7) |  |  |  |  |  | 18.8 | 978.0 | POSITIVE | 100% *A. cantonensis* | Ac13 |

TWC = total worm count, Ct = cycle threshold, SQ = starting quantity (L1s/100mg), N/A = null Ct-value (i.e. no amplification), *PCR call was positive if Ct < 35
